# Green Assessment of Sensitive Spectrofluorimetric Methods for Simultaneous Estimation of Formoterol Fumarate and Fluticasone Propionate Using a Micelle-Mediated Approach

**DOI:** 10.1007/s10895-024-04100-1

**Published:** 2025-01-16

**Authors:** Hamees A. Adawy, Maha A. Hegazy, Samah S. Saad, Shereen A. Boltia

**Affiliations:** 1 Pharmaceutical Analytical Chemistry Department, College of Pharmaceutical Sciences and Drug Manufacturing, Misr University for Science & Technology, 6th of October City, Giza, 16878 Egypt; 2 Pharmaceutical Chemistry Department, Faculty of Pharmacy, Future University in Egypt, Cairo, 11835 Egypt; 3 Analytical Chemistry Department, Faculty of Pharmacy, Cairo University, Kasr Al-Aini Street, Cairo, 11562 Egypt

**Keywords:** Fluticasone propionate, Formoterol fumarate dihydrate, B-cyclodextrin micelle, First and second derivative Spectrofluorimetry, AGREE, GAPI and RGB model

## Abstract

Highly sensitive spectrofluorimetric methods were developed for the quantitative estimation of formoterol fumarate dihydrate (FFD) and fluticasone propionate (FP) in both authentic raw materials and marketed dosage forms using a micellar-enhanced spectrofluorimetric approach. The proposed methods are based on the determination of FP in the presence of FFD using the first derivative emission spectrofluorimetry. The peak amplitude of the emission spectra of the formed micellar fluorescence was measured at 465 nm after excitation at 236 nm (λ max of FP).The second method quantifies FFD in the presence of FP using second derivative emission spectrofluorimetry. This method measures the peak amplitude of the emission spectra of the formed micelle at 283 nm, where there is no interference from FP, following excitation at 214 nm. In these methods, the emission spectra of the target drugs are measured after micellar formation, with excitation wavelengths at 214 nm for FFD and 236 nm for FP. The fluorescence intensity was enhanced using β-cyclodextrin as a micellar system after optimizing parameters affecting native fluorescence, such as diluting solvents, surfactants, and varying concentrations of β-cyclodextrin. A linear relationship was observed between the fluorescence intensity and concentration for FP over the range of 50–100 ng/mL and for FFD over the range of 30–700 ng/mL. The developed methods are simple, sensitive, non-extractive, economical, and suitable for routine analysis of FFD and FP in their raw materials and combined marketed dosage forms. The proposed techniques were carefully validated for linearity, accuracy, precision, and specificity according to the International Council for Harmonization (ICH) guidelines, demonstrating high sensitivity with lower limits of detection and quantitation. Additionally, the analytical greenness of the suggested procedures was assessed using the Analytical Greenness Metric Approach (AGREE), the Red–Green–Blue model, and the Green Procedure Analytical Index (GAPI).

## Introduction

**Fig. 1:**
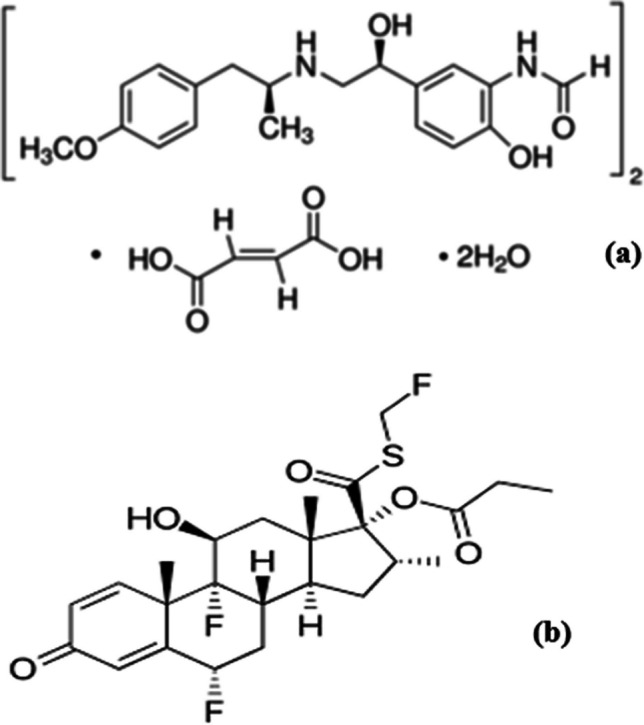
Chemical structure of (**a**) Formoterol fumarate dihydrate (**b**) Fluticasone propionate

**Formoterol Fumarate Dihydrate (FFD),** as depicted in Fig. 1a, is chemically known as N-[2-Hydroxy-5-[(1RS)−1-hydroxy-2-[[(1RS)−2-(4-methoxyphenyl)−1-methylethyl] amino]ethyl]phenyl]formamide (E)-butenedioate dihydrate [1]. It appears as a white, off-white, or slightly yellow powder, with a solubility of 0.0416 mg/mL in water and is also soluble in methanol, ethanol, and acetonitrile.

Formoterol is a long-acting beta2-adrenergic agonist (LABA), primarily used for managing asthma and chronic obstructive pulmonary disease (COPD). First approved in the U.S. in 2001, it shares similarities with salbutamol. As a beta2 agonist, it acts quickly within 2–3 min, maintaining effects for up to 12 h. Formoterol is indicated for the regular treatment of reversible airway obstruction in conditions like asthma or specific types of COPD, available as both a standalone drug or in combination with inhaled corticosteroids [2].

**Fluticasone Propionate (FP)**, illustrated in Fig. 1b, is chemically described as S-(fluoromethyl)−6α,9-difluoro-11β,17-dihydroxy-16α-methyl-3-oxoandrosta-1,4-diene-17β-carbothioate, 17-propionate. This compound is a white or nearly white powder, with limited solubility in water, partial solubility in methylene chloride, and good solubility in alcohol [2].

Fluticasone propionate is a synthetic glucocorticoid receptor agonist, containing three fluorine atoms, and is known for its anti-inflammatory and anti-allergic actions. It is commonly administered via inhalation as a powder or aerosol to prevent asthma exacerbations and manage allergic rhinitis. The drug acts primarily in the lungs, with minimal systemic absorption at typical dosages due to its low bioavailability. It is widely used for both asthma prevention and the treatment of seasonal allergic rhinitis symptoms [3].

**Flutiform®** is a pressurized inhalation suspension inhaler that combines two active ingredients: formoterol fumarate dihydrate and fluticasone propionate. This combination is used to help prevent breathing problems such as asthma and to alleviate symptoms like breathlessness and wheezing [4].

A comprehensive literature review revealed that no spectrofluorometric methods have been documented for the simultaneous determination of formoterol fumarate dihydrate (FFD) and fluticasone propionate (FP). Existing methods include only two UV spectrophotometric techniques for the concurrent analysis of FFD and FP [5, 6], as well as additional UV spectrophotometric methods for FFD or FP in combination with other drugs [7–10]. The literature also describes stability-indicating HPLC methods [11–14], HPTLC methods for FFD and FP together [15, 16], and approaches for estimating these drugs with other combinations [17–22]. However, most of these methods demand extended analysis times and the use of sophisticated equipment like HPLC and UPLC.

In contrast, this study introduces the first spectrofluorimetric methods for the simultaneous detection of FFD and FP without requiring complex sample preparation or separation. These methods offer low limits of detection and quantitation, with a detection range of 50–100 ng/mL for FP and 30–70 ng/mL for FFD. By leveraging micelle formation, the fluorescence intensity of these drugs is enhanced, making this a straightforward and cost-effective approach. Surfactants above their critical micelle concentration (CMC) effectively protect the fluorophore, reducing non-radiative energy loss. This strategy has been widely applied in conjunction with spectrofluorimetric techniques [23–25], facilitating the detection of low analyte concentrations. Cyclodextrin derivatives are also frequently employed to improve sensitivity and reduce non-radiative emissions.

The goal of this work is to develop three simple, cost-effective, and time-saving derivative spectrofluorimetric techniques that enhance the native fluorescence of FFD and FP. These methods use β-cyclodextrin (β-CD) as a fluorescence enhancer, allowing for the simultaneous determination of FFD and FP without interference from each other or any excipients. These methods are suitable for routine quality control of binary drug combinations in various matrices, including raw powders and commercial dosage forms, providing high sensitivity, precision, and accuracy.

To the best of our knowledge, no previous methods have utilized intrinsic fluorescence properties for the simultaneous spectrofluorimetric quantification of FFD and FP. Additionally, the derivative technique (D method) employed in this study helps resolve overlapping emission spectra, providing highly specific analytical signals for each drug, enabling their quantification at points where there is no contribution from the other.

Furthermore, this work evaluates the environmental impact of the proposed analytical techniques using green analytical chemistry principles, assessed through novel tools like GAPI, AGREE, and the RGB model. These tools provide a comprehensive evaluation of the environmental safety of each step, from sample collection and performance to final estimation. A color-coded system—green for low, yellow for medium, and red for high environmental influence—delivers clear quantitative data on the sustainability of the methods.

## Experimental

### Instrument

The fluorescence spectra and measurements were obtained using a JASCO FP-8200 Fluorescence Spectrometer (JASCO Corporation, Japan), equipped with a 150 W xenon lamp and a standard 1 cm path length quartz cell. Both excitation and emission monochromators had a bandwidth of 5 nm. Data acquisition was managed through the Spectra Manager® fp-8200 control software by JASCO Corporation. Additional equipment used included an ultrasonic cleaner (Branson Ultrasonic Corporation, Danbury, USA) and a vortex mixer (Lab-Line Instruments, Inc., USA).

### Materials &Reagents

**Pure Standards:** Formoterol Fumarate Dihydrate (FFD) and Fluticasone Propionate (FP) pure standards were generously provided by Novartis Company, Cairo, Egypt. Their certified purities were both 99.9%, as verified by the reported method [12].

**Chemicals:** High analytical grade sodium dodecyl sulfate (SDS), Tween 80, and β-cyclodextrin were purchased from Sigma-Aldrich.

**Reagents:** Methanol and acetonitrile of HPLC grade were obtained from Sigma-Aldrich Chemi GmbH, Germany, along with ultrapure distilled water.

***Pharmaceutical dosage form* (Flutiform® inhaler):** Batch number 9h053fc manufactured by (Fusions limited, United Kingdom, marketing authorization holder: NAPP pharmaceuticals Ltd) were used as reference product. Each metered dose (ex-valve) contains 5.0 µg of FFD and 50.0 µg of FP.

#### *Stock Standard Solutions and working standard solution*:

An accurate amount of 10 mg of FFD and FP was separately transferred into 100 mL volumetric flasks, dissolved in methanol, and diluted to the mark with the same solvent to prepare stock solutions of 100 µg/mL. These stock solutions were then further diluted with methanol to prepare working standard solutions of 1 µg/mL.

### Spectral characteristics

**Fig. 2:**
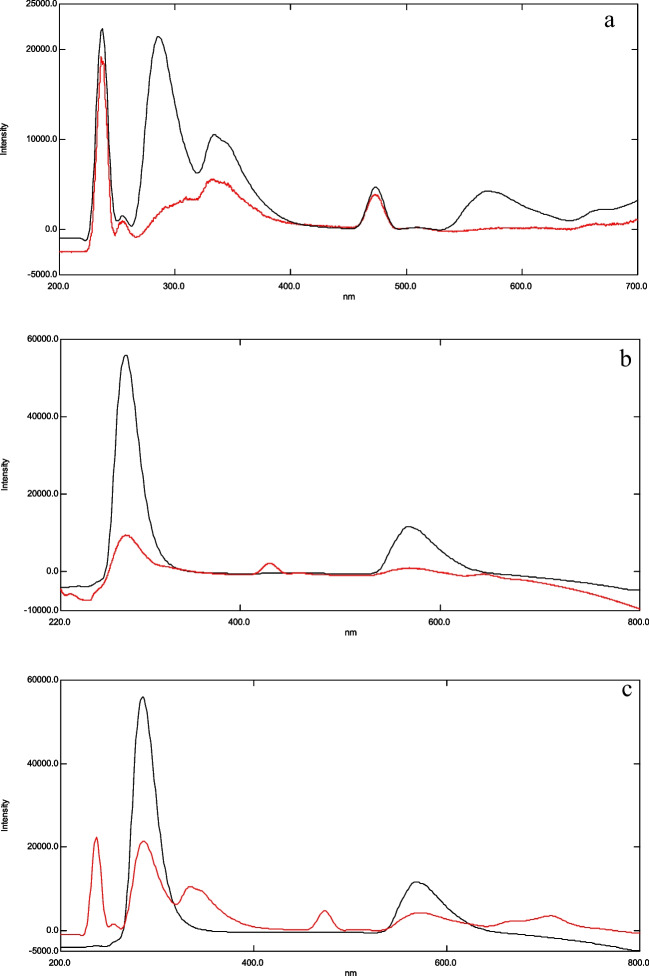
(**a**): Overlay of the fluorescence emission spectra of fluticasone propionate (50 ng/ml) after excitation at 236nm utilizing methanol as a blank (**_**) without β-cyclodextrin (**__**) after adding β-cyclodextrin surfactant. (**b**): overlay of the fluorescence emission spectra of formoterol fumarate dihydrate (50 ng/ml) after excitation at 214nm utilizing methanol as a blank (**__**) without β-cyclodextrin (_) after adding β-cyclodextrin surfactant. (**c**): Overlay fluorescence emission spectra of (50 ng.ml ^−1^) of fluticasone (**__**) and 50 ng.ml ^−1^ of formoterol fumarate (_) using methanol as blank and β-cyclodextrin as a surfactant

Aliquots equivalent to 50 ng/mL of FFD & FP were separately transferred from their stock solutions (1 µg/mL) into two 10 mL volumetric flasks and diluted to volume with methanol. The emission spectra of these solutions were then scanned after excitation at 214 nm for FFD and 236 nm for FP, using methanol as a blank, as shown in Fig. 2a and 2b. Subsequently, β-cyclodextrin was introduced as a surfactant, and the emission spectra of the 50 ng/mL solutions of FFD and FP were recorded across the 200–600 nm range. The overlay spectra were saved and are depicted in Fig. 2c.

### Procedure

#### The first derivative of zero-order emission spectra of pure Fluticasone Propionate

To quantify FP in the presence of FFD, the first derivative emission spectrofluorimetry technique was employed using the zero-order emission spectra of pure FP against the first derivative of the zero-order emission spectra of pure FFD. The peak amplitude was measured for the formed micelle at 465 nm on the D^1^ spectra, where no interference from FFD occurs.

For the analysis, aliquots ranging from 0.5 to 1 mL of FP stock solution (1 µg/mL) were accurately transferred into a series of 10 mL volumetric flasks, resulting in final concentrations of 50–100 ng.mL^−1^. To each flask, 1 mL of a 1% w/v aqueous solution of β-cyclodextrin (a non-ionic surfactant) was added, and the volume was completed with methanol to the mark.

Similarly, aliquots ranging from 0.6 to 1 mL of FFD working standard solution (1 µg/mL) were transferred into another set of 10 mL volumetric flasks, yielding final concentrations of 60–100 ng/mL. To these flasks, 1 mL of a 0.5% w/v solution of β-cyclodextrin was added, and the volume was made up with methanol.

The prepared solutions were scanned against methanol, and the first derivative curves of the zero-order emission spectra of pure FP were recorded using ∆λ = 10 and a scaling factor of 10. Similarly, the prepared FFD solutions were scanned against methanol, and the first derivative emission spectra of the zero-order were recorded. The spectra of both drugs were then overlaid. The fluorescence intensity of the peak amplitude at 465 nm in the first derivative (D1) spectra of FP was measured, showing a zero contribution for FFD.

The emission and excitation monochromatic slit widths were set at 5 nm and the data interval equal to 1 nm. All measurements were performed at room temperature using a 1 cm quartz cell.

A calibration curve was constructed by plotting the fluorescence intensity of the peak amplitude at 465 nm against the corresponding concentration of FP, and the regression parameters were computed.

#### Second derivative of zero-order emission spectra of pure Formoterol Fumarate Dihydrate

To determine Formoterol Fumarate Dihydrate in the presence of Fluticasone Propionate, the second derivative spectrofluorimetry technique was applied to the zero-order emission spectra of both pure FFD and pure FP. The fluorescence intensity at the peak amplitude of the second derivative (D^2^) spectra of FFD was measured at 283 nm, which shows a zero-crossing point for FP, indicating no interference from FP.

For this analysis, aliquots of 0.3 to 0.7 mL of FFD stock solution (1µg/mL), corresponding to 30–70 ng.mL^−1^, were accurately transferred into a series of 10 mL volumetric flasks. To each flask, 1 mL of 0.5% w/v β-cyclodextrin was added as a non-ionic surfactant, and the volume was made up to the mark with methanol. The zero-order emission spectra of pure FFD were recorded after excitation at 214 nm, followed by second derivatization.

Similarly, aliquots of 0.5 to 1 mL of FP working standard solution (1µg/mL), corresponding to 50–100 ng.mL^−1^, were accurately transferred into another set of 10 mL volumetric flasks. Each flask was then supplemented with 1 mL of 0.5% w/v β-cyclodextrin and completed to the final volume with methanol. The zero-order emission spectra of FP were recorded after excitation at 236 nm, followed by second derivatization.

A calibration curve was constructed by plotting the fluorescence intensity of the peak amplitude at 283 nm against the corresponding concentration of FFD, and the regression parameters were computed.

## Results and Discussion

Fluorescence spectroscopy is widely recognized for its significant advantages in analytical chemistry, particularly for its high selectivity and low detection limits. This method allows for the detection of trace amounts of analytes with great sensitivity, making it highly effective for the quantitative analysis of complex mixtures, such as those found in combination drug formulations, without requiring prior separation steps. This capability is particularly valuable for the analysis of marketed dosage forms, where multiple active ingredients may be present.

Moreover, the use of micelle-enhanced spectrofluorimetry further improves the technique's performance. Micellar systems can effectively reduce non-radiative decay processes by restricting the free rotational motions of fluorescent molecules, which leads to an increase in fluorescence intensity and overall sensitivity. This is because micelles provide a unique microenvironment that stabilizes the excited states of the fluorophores, minimizing quenching and enhancing signal strength.

Additionally, the micelle-enhanced approach is considered an environmentally friendly and cost-effective alternative to traditional methods. Unlike conventional organic solvents, which are often hazardous and pose significant environmental and health risks, micellar systems typically utilize surfactants that are less toxic and more sustainable. This eco-friendly aspect of micellar methods aligns with the growing demand for greener analytical techniques in the pharmaceutical industry, ensuring safety for both the analyst and the environment.

By integrating these advanced features, fluorescence spectroscopy, especially when combined with micellar enhancement, provides a robust and efficient methodology for the sensitive and selective determination of pharmaceutical compounds in various matrices, paving the way for more rapid and accurate drug analysis [26–29].

The recorded emission spectra of both of FFD and FP exhibited significant overlap, as shown in fig. 2c. This spectral overlap posed a challenge for the simultaneous analysis of the combination using direct spectrofluorimetric techniques, due to the difficulty in distinguishing the individual signals of each drug. To address this issue, derivatization spectrofluorimetry was employed. This technique enhances the resolution of overlapping spectra by transforming the zero-order emission spectra into higher-order derivatives.

Derivatization spectrofluorimetry improves the selectivity and sensitivity of the analysis by effectively narrowing spectral bandwidths and accentuating minor spectral features. By applying this technique, it is possible to determine one component of a mixture in the presence of another. This is achieved by selecting a specific wavelength where the contribution of one drug is minimal or negligible (a zero or near-zero crossing point), allowing for the accurate quantification of the other drug with a distinguishable fluorescence signal [30, 31].

The application of derivatization in spectrofluorimetry significantly enhances the analytical performance by reducing interference and improving the resolution of closely overlapping spectral bands, making it an invaluable tool for the simultaneous determination of drugs in complex mixtures.

### Parameters optimization

**Fig. 3:**
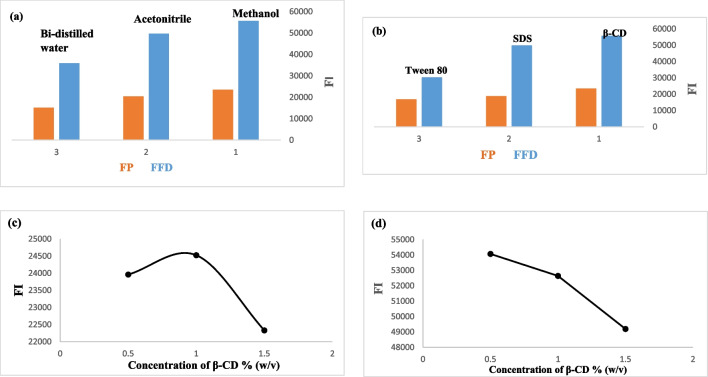
(**A**) Studying the effect of different diluting solvents at the emission fluorescence intensity of zero order spectra of pure FP (50 ng.ml^−1^) and FFD (50 ng.ml^−1^). (**b**): Studying the effect of different types of surfactants at the zero order emission fluorescence spectra of pure FP (50 ng.ml^−1^) and FFD (50 ng.ml^−1^) utilizing methanol as a blank. (**c**): Studying the effect of different β-cyclodextrin concentrations at the emission fluorescence intensity of zero order spectra of pure FP (50 ng.ml^−1^) utilizing the methanol as a blank. (**d**): Studying the effect of different β-cyclodextrin concentrations at the emission fluorescence intensity of zero order spectra of pure FFD (50 ng.ml^−1^) utilizing the methanol as a blank

Various experimental parameters that influence the native fluorescence intensity (FI) of the studied drugs were thoroughly investigated to optimize the spectrofluorimetric method. Among these parameters, the choice of diluting solvent was critical. The effects of different solvents, including methanol, water, and acetonitrile, on the native FI were evaluated, as shown in figs. 3a. The results indicated that methanol provided a significantly higher FI compared to water and acetonitrile, making it the preferred solvent for this technique due to its superior solvent properties that enhance fluorescence response.

Additionally, the impact of different types of surfactants, such as sodium dodecyl sulfate (SDS), β-cyclodextrin (β-CD), and Tween 80, figs. 3b, on the fluorescence intensity of the drugs was examined. Among these, the addition of β-cyclodextrin notably increased the FI, suggesting its effectiveness as a fluorescence enhancer. This enhancement is likely due to the ability of β-CD to form inclusion complexes with the studied drugs, reducing non-radiative decay and increasing fluorescence emission. Consequently, β-cyclodextrin was selected as the optimal fluorescence enhancer for the development of a new micellar-enhanced spectrofluorimetric method for the quantification of (FFD) and (FP) in their pure forms, laboratory-synthesized mixtures, and dosage forms.

Furthermore, the effect of varying concentrations of β-cyclodextrin, ranging from 0.5% to 1.5% w/v, on FI was studied to determine the optimal concentration for maximum fluorescence enhancement, with the most favorable results shown in figs. 3c & 3d. The optimal concentration was found to be 1% w/v, which provided the highest fluorescence intensity. This solution was prepared by dissolving 1 g of β-cyclodextrin in 100 mL of double-distilled water, ensuring consistent quality and reproducibility of the fluorescence measurements.

### The first method (D.^1^)

The study focuses on the use of first derivative spectrofluorimetry for the quantitative analysis of (FP) in the presence of (FFD) in its pure form, pharmaceutical preparations, and laboratory-synthesized mixtures. The zero-order spectra of FP and FFD overlap significantly, as illustrated in fig. 2c, which hinders the use of direct spectrofluorimetry for the determination of FP when FFD is present. To address this spectral overlap, derivative spectrofluorimetry was employed.

**Fig. 4:**
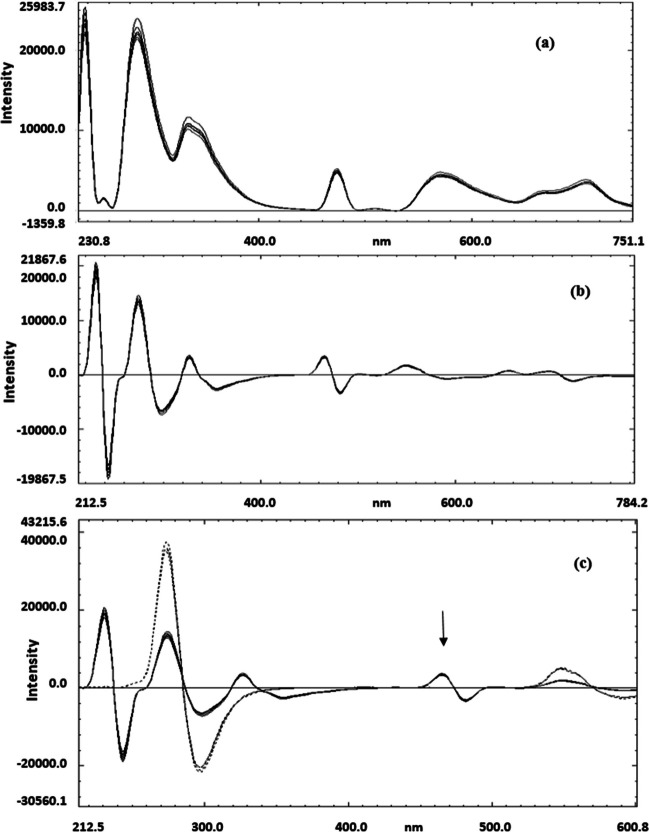
(**a**) The zero order fluorescence emission spectra of pure FP in concentration range of (50 −100 ng.ml ^−1)^ using methanol as blank and β-cyclodextrin as a surfactant. (**b**): The first derivative fluorescence emission spectra of pure FP in concentration range of (50 −100 ng.ml ^−1^) using methanol as blank and β-cyclodextrin as a surfactant. (**c**): The overlay of the first derivative fluorescence emission spectra of pure FP (__) in concentrations range of (50 −100 ng.ml ^−1)^ against first derivative fluorescence emission spectra of pure FFD (….) showing zero contribution at 465 nm using methanol as blank and β-cyclodextrin as a surfactant

The application of the first derivative technique to the zero-order emission spectra of FP (Fig. 4a) provided effective spectral resolution and yielded reproducible results, enabling the quantification of FP at 465 nm on the first derivative (D^1^) curves (Fig. 4b). This method achieved acceptable percentage recovery, with FFD showing no interference at the zero-contribution point (Fig. 4c). A calibration curve was constructed by plotting the fluorescence intensity of the peak amplitude at 465 nm against the corresponding concentrations of FP, and the regression parameters were determined. The proposed method demonstrated validity within the concentration range of 50–100 ng.ml^−1^, as shown in Table 1.

**Table 1:** Assay validation parameters of the proposed derivative spectrofluorimetric methods for the determination of a binary mixture of FFD&FP in pure powder forms

Parameters	**FP** D^1^ at 465 nm	**FFD** D^2^ at 283 nm
Linearity: range (ng. mL^−1^)	50–100	30–70
Slope	−9725.11	−92.415
Standard error of slope	134.96	1.72
Intercept	4128.9	12092
Standard error of intercept	10.38	55.37
Correlation coefficient	0.9992	0.9996
Accuracy (mean ± SD)	100.52 ± 0.91	98.96 ± 1.68
Precision: (RSD)% Repeatability ^a^	1.95%	1.92%
Intermediate precision ^b^	1.74%	1.64%
LOD ^c^	1.7	1.05
LOQ ^d^	5.19	3.17
Specificity (mean ± SD)	100.20 ± 1.83	101.69 ± 0.62

^a^ Intraday precision; average of three different concentrations of three replicate each (n = 9) repeated three times within the same day

^b^ Interday precision (n = 9); average of three different concentrations of three replicate each (n = 9) repeated on three successive days

^c^ LOD (Limit of Detection): 3.3 (SD of residual) / slope

^d^ LOQ (Limit of Quantitation):10 (SD of residual) / slope

Furthermore, it was essential to evaluate the impact of smoothing and scaling factors on the analysis. The study found that using a Δλ (wavelength interval) of 10 and a scaling factor of 10 provided optimal spectral resolution, minimized signal error, and produced well-smoothed peak shapes. Additionally, the use of β-cyclodextrin as a surfactant significantly enhanced the intrinsic fluorescence intensity by reducing the free rotational motions of molecules, thereby protecting the analyte molecules from non-radiative deactivation processes. This improvement in fluorescence intensity facilitated a more sensitive and accurate quantitative analysis of FP in the presence of FFD.

### The second method (D^2^)

**Fig. 5:**
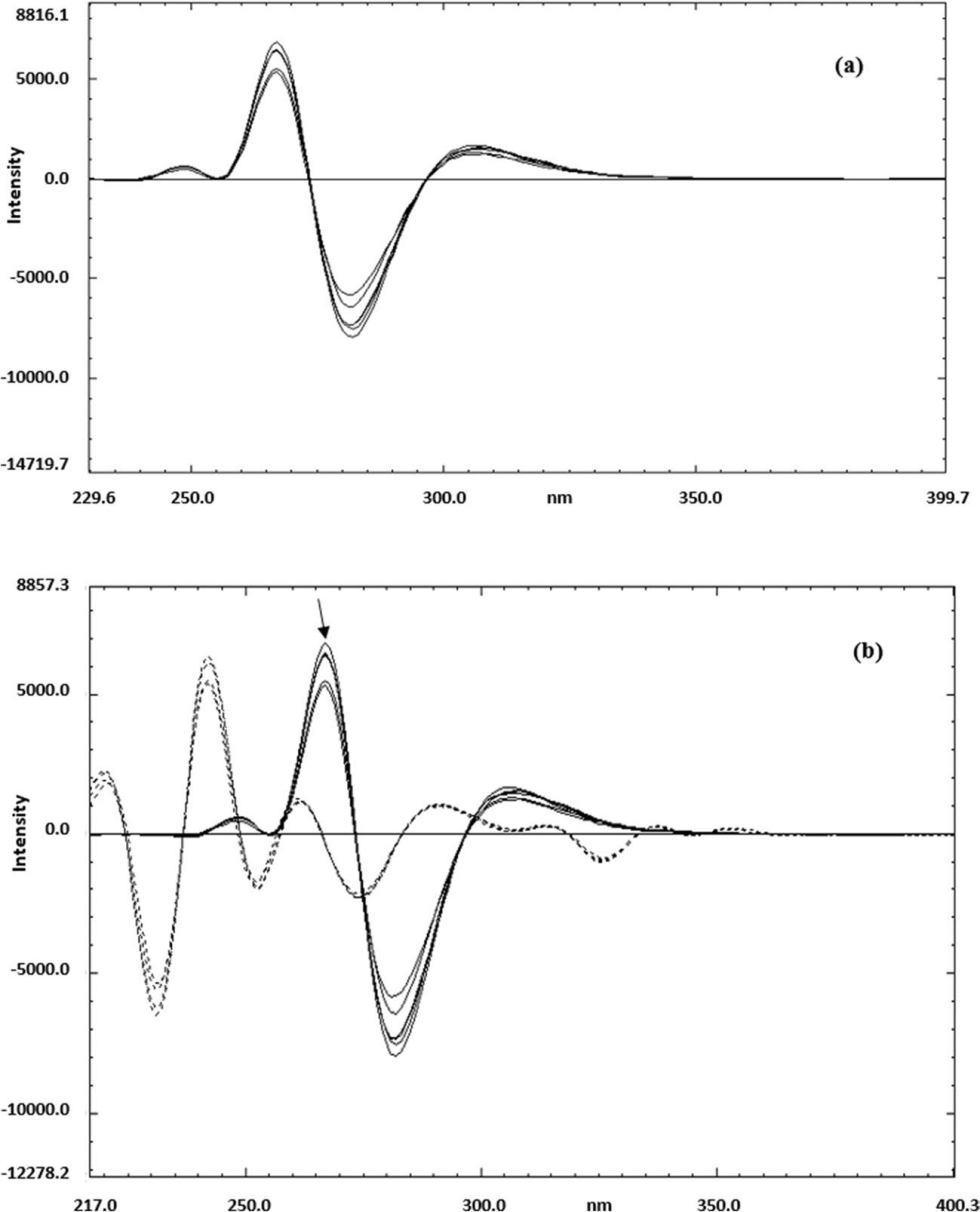
(**a**) The second derivative fluorescence emission spectra of pure FFD in concentration range of (30 −70 ng.ml ^−1^) using methanol as blank and β-cyclodextrin as a surfactant. (**b**): The overlay of the second derivative fluorescence emission spectra of pure FFD in concentration range (30 −70 ng.ml ^−1^) against the second derivative fluorescence emission spectra of pure FP (…) showing zero crossing point at 283 nm,using methanol as blank and β-cyclodextrin as a surfactant

The focus is on the quantification of Formoterol Fumarate Dihydrate (FFD) in the presence of Fluticasone Propionate (FP) using the second derivative spectrofluorimetry (D^2^). Initial attempts to determine FFD using the first derivative spectrofluorimetry (D^1^) were unsuccessful due to significant spectral overlap. Consequently, the second derivative D^2^ of the zero-order emission spectra of FFD was employed within the concentration range of 30–70 ng.ml^−1^, as depicted in Fig. 5a. This approach enabled the quantification of FFD without interference from FP at the zero-crossing point, as illustrated in fig. 5b, achieving good percentage recovery and reproducible results.

The emission spectra of pure FFD in the concentration range of 30–70 ng.ml^−1^ were recorded following excitation at 214 nm. Subsequently, the second derivative was applied to these spectra. By overlaying these second derivative spectra with those of FP in the concentration range of 50–100 ng.mL^−1^—after also applying the second derivative to FP's emission spectra—it was possible to determine FFD at 283 nm on the D^2^ spectra, with no interference from FP at the zero-crossing point.

A calibration curve was constructed by plotting the fluorescence intensity (FI) at 283 nm against the corresponding concentrations of FFD. The proposed method was validated over the concentration range of 30–70 ng.mL^−1^, with the regression parameters calculated and presented in Table 1. This method proved effective for the sensitive and selective quantification of FFD in the presence of FP.

### Assay of laboratory prepared mixtures

The proposed methods were utilized to analyze (FP) and (FFD) in their laboratory-prepared binary mixtures. For the first method, the emission spectra of the prepared mixtures at varying concentrations were recorded using methanol as a blank following excitation at 236 nm. The first derivative was then applied to these spectra to determine FP at 465 nm, yielding good recovery percentages. In the second method, the emission spectra of the laboratory-prepared mixtures were recorded after excitation at 214 nm, with methanol serving as a blank. The second derivative was applied to these spectra to determine FFD at 283 nm, resulting in acceptable recovery percentages.

The use of β-cyclodextrin as a surfactant in all the proposed methods enhanced the intrinsic fluorescence intensity of the drugs under investigation. The detailed results, including recovery percentages and other relevant data, are presented in Table 2.

**Table 2:** Determination of FP and FFD in different laboratory prepared mixtures by the suggested spectrofluorimetric techniques

Concentration ratio ng. mL^−1^ (D^1^)	Recovery % FP (D^1^)	Concentration ratio ng. mL^−1^ (D^2^)	Recovery % FFD (D^2^)
FFD + FP		**FFD + FP**	
1:10^*^	102.00	1:10^*^	102.00
1:4	100.91	1:5	102.00
1:2.33	98.50	1:2	102.12
1:1.5	101.60	1:1.5	100.61
1:1	98.00	1:14	101.72
Mean ± SD	**100.20 ± 1.83**		**101.69 ± 0.62**

^*^The concentration ratio of dosage form

### Analysis of marketed Flutiform® inhaler

Each metered dose of the inhaler contains 5.0 µg of Formoterol Fumarate Dihydrate (FFD) and 50.0 µg of Fluticasone Propionate (FP). To prepare samples for analysis, two actuations of the inhaler were discharged and dissolved in 10 mL of methanol, producing a stock solution with concentrations of 1 µg/mL for FFD and 10 µg/mL for FP.

From this stock solution, 1 mL was transferred to a 10 mL volumetric flask and diluted with methanol to obtain a solution with concentrations of 0.1 µg/mL for FFD and 1 µg/mL for FP. A further dilution was performed by transferring 1 mL of the prepared solution into another 10 mL volumetric flask and diluting with methanol to achieve a final concentration of 0.1 µg/mL for FP. The practical concentration of FP was then calculated by substituting the fluorescence intensity at the peak amplitude of 465 nm into the corresponding regression equation. The recovery percentages obtained were acceptable, as verified using the developed methods for laboratory-synthetic mixtures, which utilized β-cyclodextrin as a surfactant and methanol as a blank.

For FFD quantification, a 5 mL aliquot of the 0.1 µg/mL FFD solution was transferred to a 10 mL volumetric flask and diluted to the mark with methanol, resulting in a final concentration of 0.05 µg/mL. The propellant was allowed to evaporate prior to analysis. The concentration of FFD was determined using the second spectrofluorimetric procedure described for laboratory-synthetic mixture analysis. The calculated concentrations demonstrated acceptable recovery percentages, as summarized in Table 3.

**Table 3:** Determination of FFD and FP in Flutiform ® inhaler by the proposed derivative spectrofluorimetric methods

Preparation	Claimed Concentration (ng. mL^−1^)	FP (Recovery % ± SD^*^)	FFD (Recovery % ± SD^*^)
	FFD + FP	D^1^ at 465 nm	D^2^ at 283 nm
Flutiform ®Inhaler B.N 9h053fc	50 + 100	100.11% ± 1.05	101.2% ± 0.94

^*^Average of three determinations

### Assessment of the Greenness Profile of Analytical Methods

Green Analytical Chemistry (GAC), introduced in 2000, aims to reduce or eliminate the negative impacts of analytical processes on both analysts and the environment. Striking a balance between enhancing the environmental sustainability of analytical methods and maintaining the quality of their outcomes is a complex task. Consequently, achieving a suitable equilibrium between analytical performance and expected greenness is both motivating and challenging, often complicating the evaluation process.

### Green Analytical Procedure Index (GAPI)

GAPI is a recently developed tool that provides semi-quantitative data, allowing researchers to evaluate the environmental sustainability of analytical methods based on their criteria. This tool combines features of the Eco-scale and NEMI (Płotka-Wasylka, 2018) and generates a unique pictogram that visually ranks the environmental impact of each step in the analytical process. It uses five pentagrams to assess and measure the ecological footprint of each stage, with color codes: green for low impact, yellow for medium impact, and red for high impact. Each section of the pictogram represents a different aspect of the analytical procedure, such as sample collection and preparation, waste management, instrumentation, and the safety of reagents and chemicals, including their health impacts. Fields turn green when specific requirements are met, making GAPI more advantageous compared to traditional tools like NEMI, which only indicates whether a risk falls below or above a set threshold [32].

**Fig. 6:**
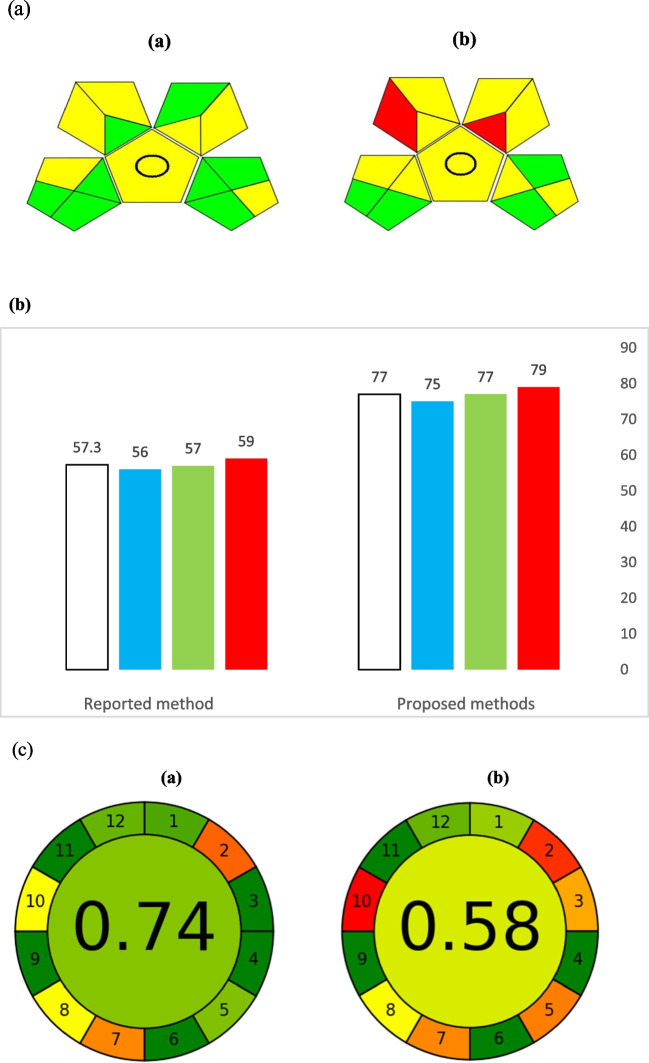
(**a**) GAPI assessment pictogram generated for the evaluation of proposed spectrofluorimetry method's greenness (**a**) and the HPLC reported method (**b**).^.^(**b**) the comparison between the average (CS %) results obtained from RGB model for the developed spectrofluorimetric methods and reported HPLC method. (**c**): the results of AGREE assessment evaluation of GAC aspects of the proposed spectrofluorimetric methods (**a**), and the reported HPLC (**b**)

Figure 6a illustrates the application of GAPI to our proposed spectrofluorimetric methods. For sample preparation, the method aligns with sustainable practices: no preservatives were added, no transport system was required, and no extraction was involved—indicating a straightforward direct-type method. Concerning instrumentation, the energy consumption per sample was less than 0.1 kWh [33], occupational hazards were minimized with hermetic sealing, and waste generation was kept below 10 mL. The reagents used ranged from 10 to 100 mL (indicating a moderate impact). A review of the safety material data sheets (MSDS) showed that methanol, one of the solvents used, had three hazard pictograms, while beta-cyclodextrin was considered non-hazardous, with no associated hazard symbols.

### RGB Model (Red, Green, and Blue)

The RGB model is named after the three primary colors—Red, Green, and Blue—which represent key aspects of any analytical technique. In this model, red corresponds to analytical performance, which is assessed through standard validation procedures. Green represents safety and environmental sustainability, including aspects of Green Analytical Chemistry (GAC) such as waste and reagent hazards, occupational risks, and energy consumption. Blue relates to productivity and practical efficiency, covering factors like sample preservation, methodological simplicity, cost and time effectiveness, and the frequency of instrument maintenance [34].

Each aspect is quantitatively evaluated using a Color Score (CS) ranging from zero to one hundred percent. A method is considered to have reached a primary color if its corresponding CS is 66.6% or higher, which is termed the "satisfaction range." Conversely, if the CS is below 33.3%, it falls within the "tolerance range," indicating the method does not fully achieve that color.

Proper implementation of GAC principles is demonstrated by minimizing reagent use, selecting low-toxicity reagents, conserving energy, reducing waste, and eliminating hazards to analysts. After evaluating all aspects, the final color is white, representing an integrated overall performance score calculated using an Excel worksheet. The model’s brilliance value, which combines the criteria of all colors, is quantitatively determined. Figure 6b shows the average CS results for the three proposed spectrofluorimetric methods compared to those of the reported HPLC method [12].

### AGREE: Analytical Greenness Metric Approach

The AGREE tool, also known as the Analytical Greenness Calculator, evaluates the environmental sustainability of analytical procedures based on the 12 principles of Green Analytical Chemistry, which are transformed into a unified 0–1 scale [35]. The final score is determined by evaluating the performance significance of the analytical procedures, resulting in a clock-like pictogram that provides a comprehensive, informative, and sensitive assessment of the method’s greenness.

Since defining and assessing greenness can be complex, the metric system incorporates multiple aspects to capture a full picture of environmental impact. Input criteria cover material requirements (quality and quantity), waste generation, energy consumption, analyst safety, and overall procedural approach, including factors like pre-treatment steps and the location of the analytical device relative to the object of investigation.

The software is user-friendly, allowing easy assessment with the final score and color-coded representation displayed at the center of the pictogram. Scores closer to 1 indicate high greenness, while those near 0 suggest low greenness in terms of analytical performance. Each segment of the pictogram reflects specific assessment criteria, marked by colors and corresponding numbers, including the number of samples and sample sizes, pre-treatment requirements, level of miniaturization and automation, analyte throughput, procedural steps, reagent quantities and associated hazards, and energy consumption of the instrumentation. These aspects were evaluated using the AGREE tool, and the results are displayed in fig 6c.

### Method validation

The proposed methods were validated according to the International Council for Harmonization (ICH) guidelines [36], focusing on several key parameters: linearity, range, accuracy, precision, specificity, limit of detection (LOD), and limit of quantification (LOQ).

### Linearity

The linearity of the proposed methods was assessed by analyzing a series of concentrations for Fluticasone Propionate (FP) and Formoterol Fumarate Dihydrate (FFD). The concentration ranges were 50–100 ng.ml^−1^ for FP and 30–70 ng.mL^−1^ for FFD. Emission spectra were recorded over a wavelength range of 200–600 nm using methanol as a blank and β-cyclodextrin as a surfactant. The calibration curves showed a linear response within these concentration ranges, as indicated in Table 1.

### Accuracy

To evaluate the accuracy of the proposed methods, different concentrations of (FFD) formoterol fumarate dihydrate (35, 45, 55, and 65 ng/mL) and (FP) Fluticasone propionate (55, 75, 85, and 95 ng/mL) within their respective linearity ranges were analyzed. The recovery percentages were calculated for each concentration using the corresponding regression equations, demonstrating high accuracy, as shown in Table 1.

### Precision

#### Repeatability

The repeatability of the methods was assessed by analyzing three concentrations of FFD (30, 50, and 70 ng.mL^−1^) and FP (60, 70, and 90 ng.mL^−1^) three times within the same day. The relative standard deviation (RSD%) values were calculated, confirming good repeatability (Table 1).

### Intermediate-Precision

To evaluate intermediate precision, the same concentrations of FFD and FP were analyzed three times over three successive days. The calculated RSD% values confirmed the robustness and consistency of the methods over time (Table 1).

### LOD and LOQ

LOD and LOQ were calculated from the standard calibration curves. The LOD and LOQ values were derived using the formulae:

- LOD = (3.3 × SD) / S
- LOQ = (10 × SD) / S

where SD is the standard deviation of the y-intercepts of the regression lines, and S is the slope of the calibration curve. The results are presented in Table 1.

### Specificity

The specificity of the methods was tested using laboratory-prepared mixtures containing various ratios of FP and FFD within their linearity ranges and at the dosage form ratio. Recovery percentages and RSD% values were calculated, demonstrating high specificity with minimal interference from excipients (Table 2). The validity of the methods was further confirmed using the standard addition technique, as shown in Table 4.

**Table 4:** Application of standard addition technique for the determination of FP &FFD in pharmaceutical dosage form by the suggested derivative spectrofluorimetric techniques

	Standard addition for FP			Standard addition for FFD		
pharmaceutical preparation	Added FP (ng.ml^−1^)	Taken FP (ng.ml^−1^)	Recovery % D^1^ at 465 nm	Added FFD (ng.ml^−1^)	Taken FFD (ng.ml^−1^)	Recovery % D^2^ at 283nm
	20	40	98.00	10	20	101.99
Flutiform ®	30	40	102.00	20	20	102.00
Inhaler	40	40	98.12	30	20	99.27
B.N 9h053fc	50	40	98.42	40	20	100.72
	60	40	98.20	50	20	99.63
Mean			**98.95**			**100.72**
SD			**1.70**			**1.18**
RSD%			**1.71**			**1.19**

This comprehensive validation procedure confirms that the proposed methods meet stringent criteria for high accuracy, precision, specificity, low detection, and quantification limits, along with acceptable linearity and a wide range.

### Statistical Comparison

The results obtained from the proposed spectrofluorometric methods were statistically compared with those from a previously reported HPLC method [12]. The comparison was conducted using the student’s t-test and F-test, which revealed no significant differences between the methods (Table 5).

**Table 5:** Statistical analysis of the results obtained by the proposed derivative spectrofluorimetric methods and the reported HPLC method for the simultaneous determination of FFD & FP

	**FP**		**FFD**	
Parameters	D^1^ at 465 nm	Reported method ^b^	D^2^ at 283 nm	Reported method ^b^
Mean R%	99.95%	99.6%	100.5%	99.5%
SD	0.85	0.66	1.19	1.26
n	4	4	4	4
Variance	0.73	0.44	1.43	1.60
Student’s t- test ^a^	0.92 (1.94)	-	0.08 (2.77)	-
F –test ^a^	0.48 (0.16)	-	6.40 (9.27)	-

^a^ Tabulated t-test and F values was obtained at P = 0.05

^b^ Reported RP-HPLC method using C18 column, acetonitrile: 0.01 m ammonium

dihydrogen o-rtho phosphate buffer (80:20) v/v as a mobile phase and UV detection at 215 nm [12]

Additionally, the ability of the proposed methods to determine FP and FFD was further validated by subjecting the results to statistical analysis using a one-way ANOVA test. This analysis confirmed no significant differences among all proposed methods, highlighting their reliability and consistency (Table 6).

**Table 6:** Results of One -way ANOVA for comparison of the proposed procedure results for the determination of FP and FFD and the reported method [12]

	Source of variation	DF	Sum of squares	Mean square	F -value	P -value	F crit
FP	Between groups	1	0.002	0.002	0.0019	0.96	5.32
	Within groups Total	8 9	9.07 9.072	1.13			
FFD	Between groups	2	1.61	0.80	0.68	0.52	3.88
	Within groups	12	14.17	1.18			
	Total	14	15.78				

## Conclusion

The current study focuses on enhancing the intrinsic fluorescence properties of the investigated drugs. The fluorescence spectrum of a molecule is influenced by its ability to absorb electromagnetic radiation, and the addition of a surfactant can significantly increase fluorescence intensity through the micelle enhancement effect. This effect helps reduce non-radiative deactivation processes, thereby boosting the fluorescence signal. The methods adopted in this study are both sensitive and straightforward, offering reproducible results for the determination of the target drugs at very low concentration levels using sensitive derivative emission spectrofluorimetry techniques.

Derivative spectrofluorimetry techniques provide several advantages: they are more sensitive than other detection systems like classical UV absorption, less costly compared to LC–MS/MS detection, and more time-efficient than HPLC. The use of derivative techniques in this context enhances the selectivity of drug analysis in the presence of another combined drug, effectively cancelling out the spectrum of the interfering substance and increasing the sensitivity of the analytical process.

Another significant advantage of the proposed methods is their environmentally friendly nature. The techniques avoid the use of hazardous organic solvents or compounds, making them green and sustainable. Furthermore, these methods can be readily applied in quality control laboratories for the simultaneous analysis of the investigated drugs in pure powder form, laboratory-synthesized mixtures, and marketed dosage forms without the need for prior separation steps or sophisticated instruments. This versatility and simplicity underscore the practical utility of the developed methods in various analytical settings.

## Data Availability

The datasets used and/or analyzed during the current study are available from the corresponding author on reasonable request.
